# RpaA Regulates the Accumulation of Monomeric Photosystem I and PsbA under High Light Conditions in *Synechocystis* sp. PCC 6803

**DOI:** 10.1371/journal.pone.0045139

**Published:** 2012-09-14

**Authors:** Waqar Majeed, Yan Zhang, Yong Xue, Saurabh Ranade, Ryan Nastashia Blue, Qiang Wang, Qingfang He

**Affiliations:** 1 Department of Applied Science, University of Arkansas at Little Rock, Little Rock, Arkansas, United States of America; 2 High Tech Research Center, Shandong Academy of Agricultural Sciences, Jinan, Shandong Province, China; 3 Institute of Hydrobiology, Chinese Academy of Sciences, Wuhan, Hubei Province, China; University of Hyderabad, India

## Abstract

The response regulator RpaA was examined by targeted mutagenesis under high light conditions in *Synechocystis* sp. PCC 6803. A significant reduction in chlorophyll fluorescence from photosystem I at 77 K was observed in the RpaA mutant cells under high light conditions. Interestingly, the chlorophyll fluorescence emission from the photosystem I trimers at 77 K are similar to that of the wild type, while the chlorophyll fluorescence from the photosystem I monomers was at a much lower level in the mutant than in the wild type under high light conditions. The RpaA inactivation resulted in a dramatic reduction in the monomeric photosystem I and the D1 protein but not the CP47 content. However, there is no significant difference in the transcript levels of *psaA* or *psbA* or other genes examined, most of which are involved in photosynthesis, pigment biosynthesis, or stress responses. Under high light conditions, the growth of the mutant was affected, and both the chlorophyll content and the whole-chain oxygen evolution capability of the mutant were found to be significantly lower than those of the wild type, respectively. We propose that RpaA regulates the accumulation of the monomeric photosystem I and the D1 protein under high light conditions. This is the first report demonstrating that inactivation of a stress response regulator has specifically reduced the monomeric photosystem I. It suggests that PS I monomers and PS I trimers can be regulated independently for acclimation of cells to high light stress.

## Introduction

Light is the ultimate energy for photosynthesis; however, excess excitation energy as a result of high light (HL) illumination can damage photosynthetic cells [1]–[5]. Photosynthetic organisms have evolved various mechanisms to acclimate to HL stress through altering the photosynthetic apparatus. These mechanisms include changes in the reaction center pigment-protein complexes [6], state transitions [7]–[9], and stabilization of photosynthetic membranes [5], [10]. The energy transfer between photosystems in cyanobacteria is regulated in a light-dependent manner where the photosystems undergo rapid adjustments to balance light absorption. The regulation of the photosytem I (PS I) and/or PS II content or the PS I to PS II ratio in response to changing light conditions is arguably one of the most critical processes in HL acclimation [10]–[15]. The PS I to PS II ratio in cyanobacteria decreases upon shift to HL due to suppression in the amount of functional PS I [13], [16]. The more prominent decrease in PS I content than the PS II results in a decrease of the PS I to PS II ratio under HL conditions. This process is triggered by the energy coupling between phycobilisome (PBS) and photosystems in response to varying light conditions. Most likely, a highly developed fabric of gene regulatory systems plays the key role in photoacclimation and survival in the ever-changing light environments. For example, PmgA has been reported to be responsible for the down-regulation of PS I under HL conditions [13], [17]; and the DspA protein (or Hik 33) has been reported to be responsible for transcriptional regulation of stress response and photosynthetic genes including PS I [18].

The PS II reaction center is the primary target of the photoinhibition that is characterized by the damage to the D1 protein (encoded by the *psbA* genes) as a consequence of excess excitation [19]–[22]. The rapid restoration of PS II function following photoinhibition requires degradation of the damaged D1 polypeptide, *de novo* synthesis of D1 polypeptide, and incorporation of a new D1 copy into the PS II complex [20], [22].

In cyanobacteria, PBS serves as the light-harvesting antenna for transfer of light energy to PS I and PS II [23]. PBS consists of over 100 polypeptides which constitutes the extrinsic membrane complex, and, due to its high mobility, PBS allows for the redistribution of excitation energy between the two photosystems [24] through a direct interaction with either PS I or PS II [23]–[25]. It has been reported that RpaA, a regulatory protein, regulates the energy transfer from PBS to PS I [26], [27]. The RpaA deletion mutant maintains the intact PBS core composition. However, the efficiency of energy transfer from PBS to PS I was reduced, in favor of energy transfer to PS II [27]. RpaA has also been reported as a part of a two-component regulatory system (the DspA-RpaA system), regulating the expression of genes in response to hyperosmotic stress [28]. Recently, RpaA has been reported to be involved in KaiC mediated circadian clock, and the SasA-RpaA two-component regulatory system regulates the circadian timing from posttranslational oscillation to the transcriptional machinery [29]. The intricate interplay among the systems that regulate the expression of the photosynthetic genes in response to HL or other stress conditions is yet to be elucidated.

In this work, we characterized a fully segregated RpaA inactivated mutant of the cyanobacterium *Synechocystis* sp. PCC 6803. Although RpaA has been implicated in regulation of energy transfer from PBS to PS I, our aim was to investigate the role of RpaA in regulation of photosynthetic assembly under HL conditions. This is due to the fact that RpaA constitutes a two component regulatory system with DspA, a global regulator that controls sets of photosynthetic and HL responsive genes [18], [30]. We found that the RpaA inactivation resulted in a significant reduction in chlorophyll fluorescence from PS I at 77 K in the whole cell under HL conditions. However, the level of chlorophyll fluorescence emission from the PS I trimers at 77 K are similar to that of the wild type (WT), while the chlorophyll fluorescence from the PS I monomers was at a much lower level in the mutant as compared with the WT under HL conditions. The RpaA inactivation also caused a dramatic reduction in the monomeric PS I and the D1 protein but not the CP47 content. Therefore, the RpaA inactivation specifically impacted the accumulation of the D1 protein rather than affecting PS II as a whole. In contrast, there is no significant difference in transcript levels of all genes examined, most of which are involved in photosynthesis, pigment biosynthesis, and stress responses. Furthermore, the chlorophyll content and the whole-chain oxygen evolution capacity of the mutant were significantly reduced when the cells were exposed to prolonged periods of high intensity light. The mutant also showed retarded growth under HL conditions. Therefore, RpaA regulates (perhaps posttranscriptionally) the accumulation of monomeric PS I and the D1 protein, and it is important for photoacclimation under HL conditions.

## Results

### RpaA Inactivation Affects Cell Growth

**Figure 1:**
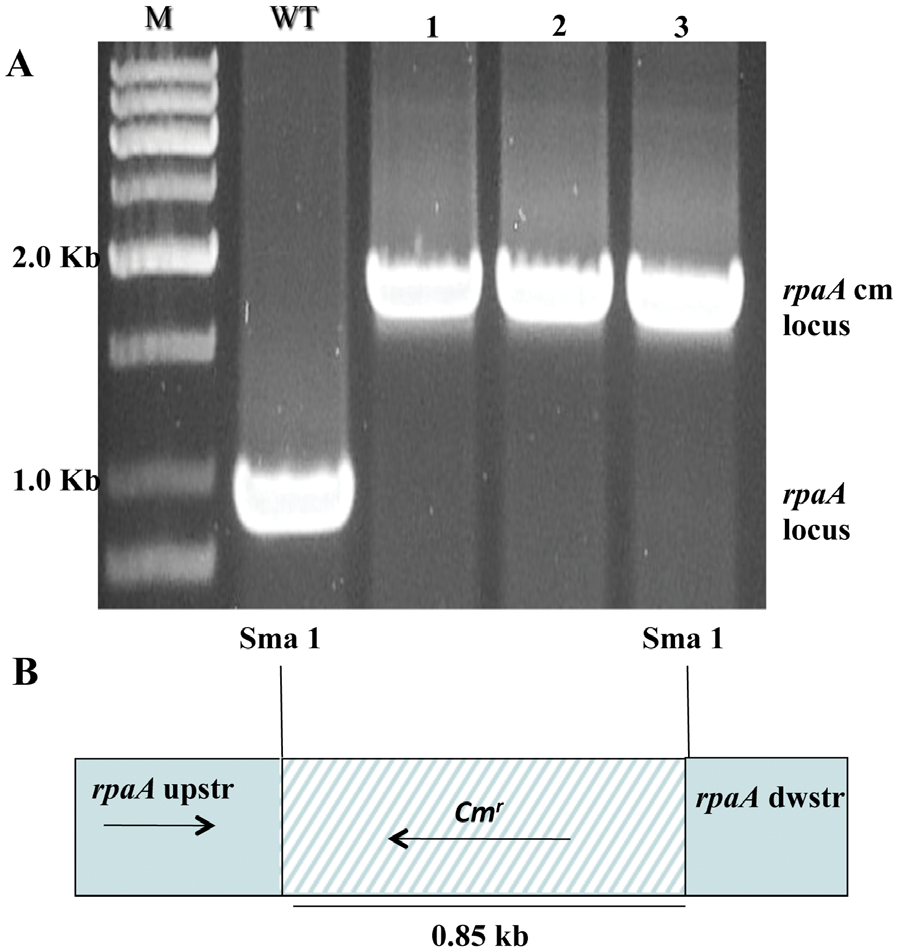
Inactivation of RpaA. **A**. PCR analysis of the *rpaA* loci. Genomic DNA template was isolated from the putative RpaA inactivation mutants (lane 1, 2 and 3) and WT cells. **B,** Depiction of the plasmid construct used to generate the RpaA inactivation strains. M, DNA size marker; WT, wild type.

**Figure 2:**
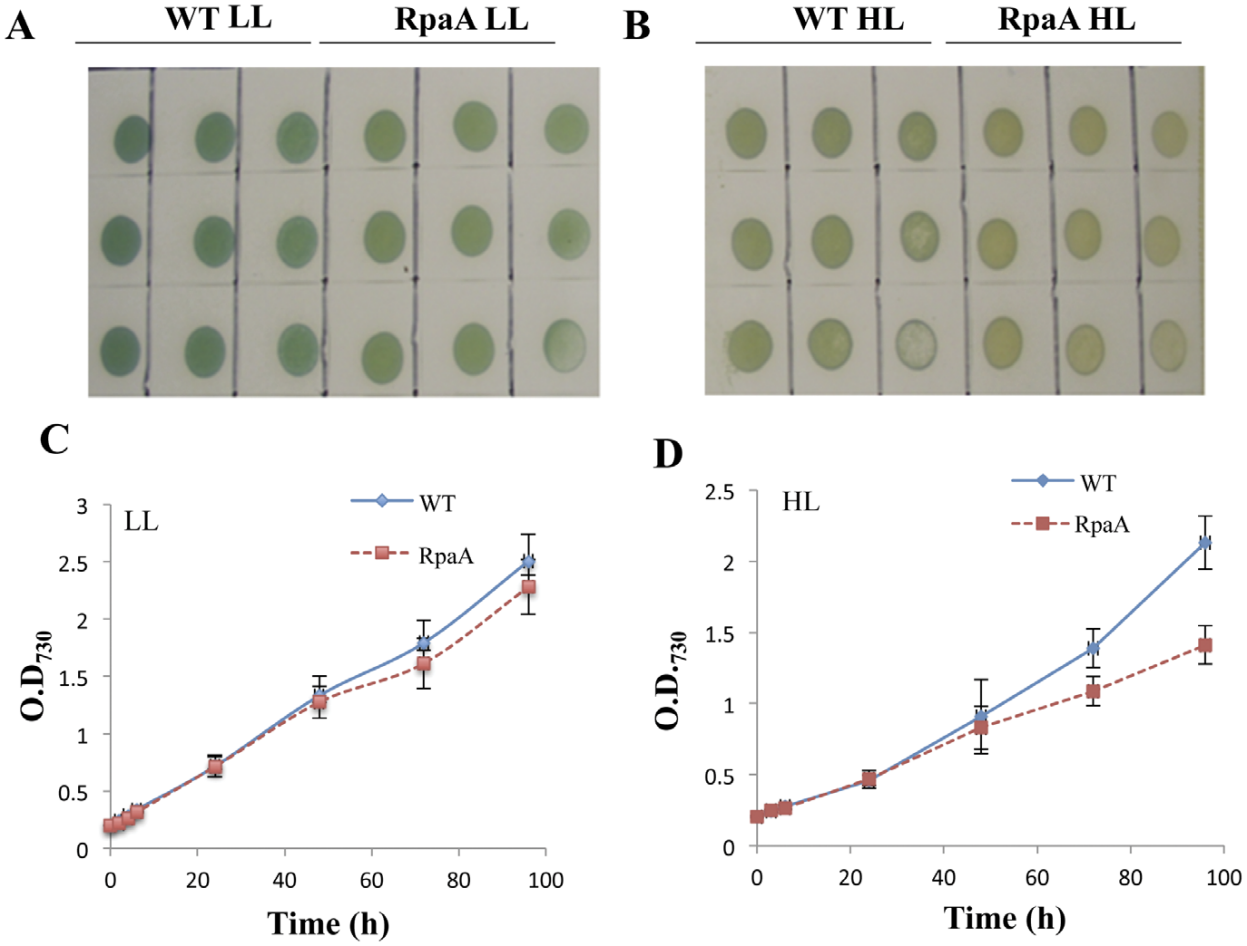
Growth of WT and the RpaA inactivation mutant under LL and HL conditions. A and **B**, WT and the mutant were grown on BG-11 agar plate in LL (**A**) or under HL conditions (**B**) for 5 days. Growth of the cells were also monitored in liquid BG-11 medium as a change in optical density at 730 nm for 4 days under LL (C) and under HL conditions (D). Curves were generated by averaging the data obtained from three representative experiments on different days.

The *rpaA* gene was inactivated from WT, *Synechocystis* sp. PCC 6803, by target mutagenesis, and homoplasmic mutants were obtained (Fig. 1). The RpaA inactivated mutant did not show any significant defect in growth under standard growth conditions (in BG 11 medium at 30°C with 50 µmol of photon m^−2 ^s^−1^) (Fig. 2A&C). However, the mutant cells appeared to be somewhat bleached and turned orange under HL conditions (Fig. 2B). The mutant exhibited retarded growth under HL conditions (400 µmol of photon m^−2 ^s^−1^ at 30°C), as compared with the WT (Fig. 2D).

### RpaA Inactivation Reduces Chlorophyll Content and Oxygen Evolution Capacity of HL-grown Cells

**Figure 3:**
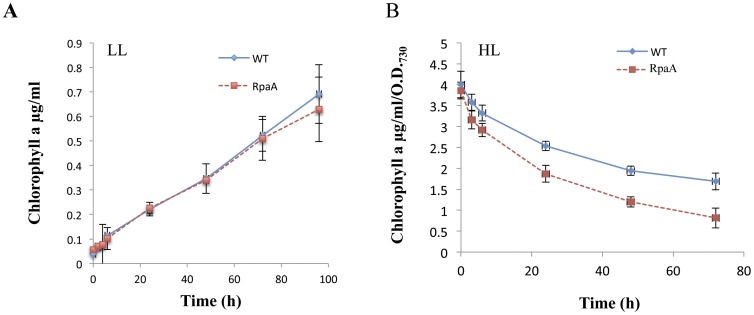
Total chlorophyll levels in the cells of WT and the RpaA mutant. Total chlorophyll content was measured in cells grown in LL and HL for various lengths of time as indicated. Chlorophyll is extracted in dimethylformamide and quantified using spectrophotometer. Data were derived from four independent experiments on different days.

The pigment composition of the cells was analyzed under low and HL conditions. No difference in chlorophyll content was observed under LL condition, while, the chlorophyll content in both WT and the mutant decreased as HL exposure proceeded, but the reduction in the chlorophyll content was aggravated in the mutant (Fig. 3B). The mutant retained only half of the chlorophyll as compared to WT upon exposure to HL for 72 hours.

Photosynthetic electron transport activity was evaluated by measuring the O_2_ evolution capacity in the absence of electron donor (whole chain activity) of WT and the mutant cells grown under both normal growth light and HL conditions. The results are shown in Table 1. The oxygen evolution capacity of the RpaA inactivated mutant is somewhat higher than that of the wild type when cells were grown in low light, but the difference is relatively small. In contrast, the mutant evolved significantly lower amounts of oxygen as compared with WT when cells were grown in HL for 48 hours. Clearly, the RpaA inactivation affects the whole chain electron transport activity under HL conditions.

**Table 1:** Oxygen evolution of WT and the RpaA mutant.

Strain	Whole chain oxygen evolution (µmolO_2_/mg chl/h)	
	LL	HL
WT	149±18	331±37
RpaA	161±19	225±25

Cells were grown in LL or HL for 24 h in BG-11 medium. The whole-chain oxygen evolution (µmol O_2_/mg Chl/h) was measured using a Clark-type oxygen electrode. Data were generated by averaging four independent experiments on different days.

LL-Low light; HL-High light.

### RpaA Inactivation does not Affect Transcription of Genes Involved in Photosynthesis, Pigment Biosynthesis, and Stress Responses under HL Conditions

**Figure 4:**
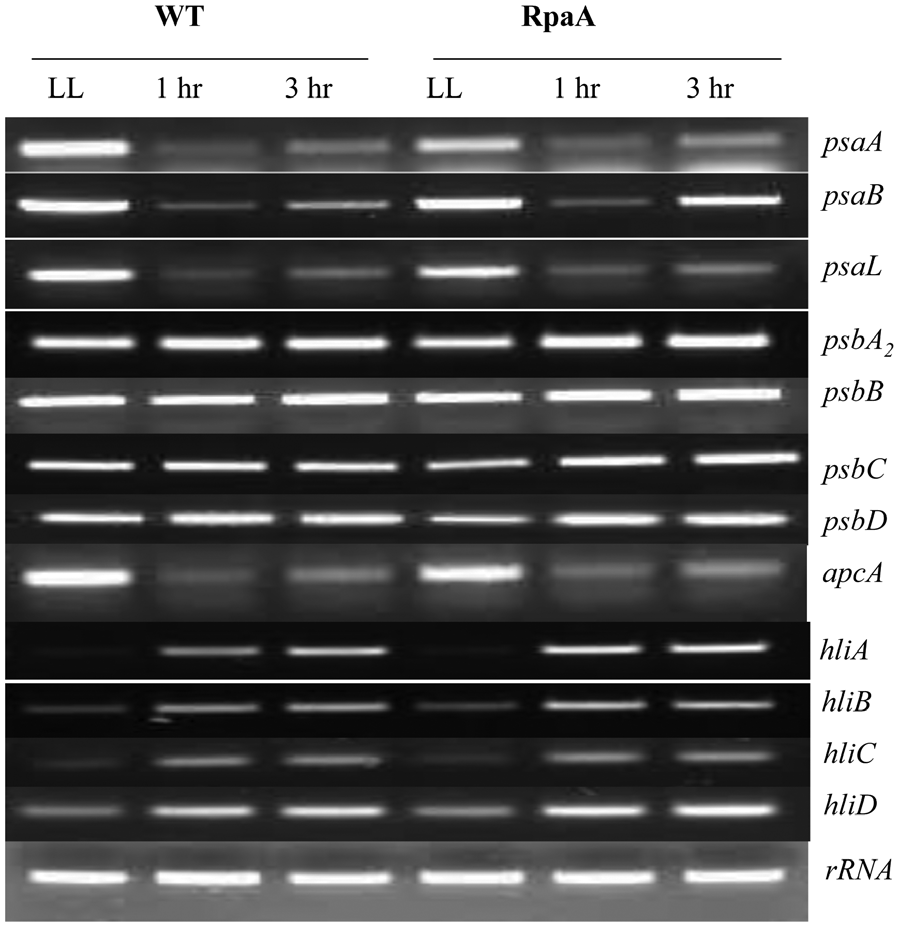
RT-PCR analysis of WT and the RpaA inactivation mutant. Total RNA was isolated from cells grown in LL or treated with HL for 1 or 3 h. cDNA was prepared from total RNA. Gene specific primer for *psaA*, *psaB, psaL, psbA_2_*, *psbB*, *psbC*, *psbD,apcA, hliA*, *hliB*, *hilC*, *hliD* were used for PCR.

Transcriptional regulation is one of the most important mechanisms in regulation of responses to various stimuli perceived by cyanobacteria. Upon exposure to stress conditions such as high intensity light, *Synechocystis* cells accumulate, for example, *hli* transcripts encoding the high light inducible polypeptides (HLIP) [5] while the *psaA* gene down-regulates [31]. Here we examined the impact of the RpaA inactivation on transcript accumulation of a number of genes involved in photosynthesis, pigment biosynthesis, and stress responses. We did not observe significant difference in transcript levels of *psaA*, *psaB, psaL, psbA_2_*, *psbB, psbC, psbD, apcA, hliA*, *hliB*, *hliC*, *hliD* either on brief exposure to HL or on prolonged exposure as compared with WT (Fig. 4). These data suggest that RpaA may not play a key role in the transcriptional regulation of *psaA*, *psaB, psaL, psbA_2_*, *psbB, psbC, psbD, apcA, hliA*, *hliB*, *hliC*, *hliD* genes in response to HL.

### RpaA Inactivation Affects Photosystem Stoichiometry and Photosystem Activity

**Figure 5:**
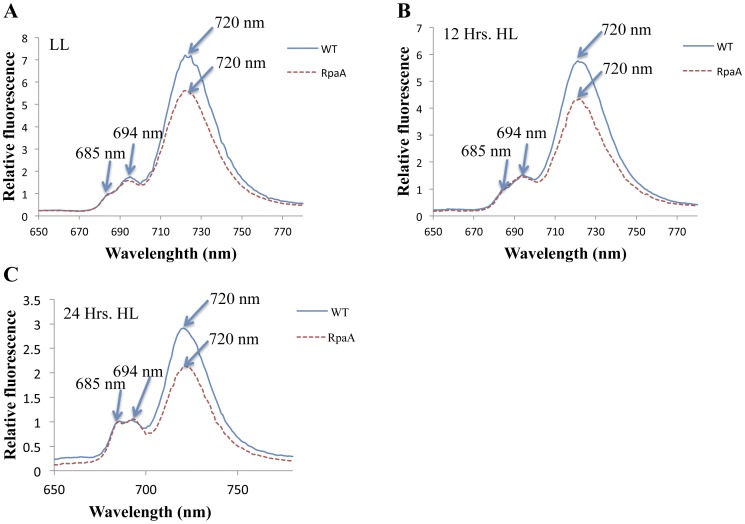
77 K fluorescence of whole cells of WT and the RpaA inactivation mutant. A, Spectra were recorded from cells grown in LL (A), 12 h and 24 h after exposure to HL. The fluorescence emission spectra were recorded at an excitation wavelength of 430 nm at 77 K. All samples correspond to 15 µg of chlorophyll/ml. The spectra were normalized at 685 nm.

The impact of the RpaA inactivation on stoichiometry of photosystems was diagnostically investigated by 77 K fluorescence spectroscopy (Fig. 5). WT and mutant cells were grown under LL and HL conditions, and their chlorophyll fluorescence emission spectra were recorded in liquid nitrogen. Spectra were recorded at three different time points, before the cells were exposed to HL and at 12 and 24 hours after cells were transferred to HL. Cells showed a major fluorescence emission peak at 720 nm, which corresponds to PS I-associated chlorophyll, and two smaller peaks at 685 nm and 694 nm, which originated mainly from PS II associated chlorophyll. The relative fluorescence of PS I chlorophyll (*F*_PSI_) of the mutant strain was significantly lower than that of WT when normalized at 685 nm (Fig. 5). In cyanobacteria, the *F*_720_ to *F*_685_ ratio is generally believed to correlate with the relative content of PS I and PS II [14]. The mutant cells have a lower PSI to PSII ratio (Table 2). The lowered *F*_720_ to *F*_685_ ratio seen in the mutant suggests that the RpaA inactivation affected the stoichiometry of the photosystems.

**Table 2:** PS I to PS II ratio the whole cells of WT and the RpaA mutant.

	*F_720_/F_685_*		
	LL	12 Hrs	24 Hrs
WT	6.92±0.31	5.67±0.26	2.91±0.16
RpaA	5.41±0.28	4.29±0.32	2.10±0.23

WT and mutant cells were grown in LL and then transferred to HL for 24 h. Whole cells were analyzed by fluorescence emission spectroscopy at 77 K with an excitation wavelength of 430 nm. Spectrum were recorded at three different time points, first before the cells were transferred to HL then 12 h and 24 h post transfer to HL respectively. The ratio of the fluorescence intensity originated from PS I (720 nm) and PSII (685 nm) was used as an estimate of PS I to PS II ratio. Data were generated by averaging three independent experiments on different days.

We also evaluated the PS I and PS II activities in mutant cells. The mutant cells grown in both LL and HL were found to exhibit lower PS I activities as compared to WT. However, the impact of RpaA deletion on PS I activity is more prominent (Table 3). In contrast, the PS II activities of the mutant cells are comparable to WT level.

**Table 3:** PS I and PS II activities of WT and the RpaA mutant.

Strain	PS I		PS II	
	LL	HL	LL	HL
WT	143±29	101±15	127±17	142±23
RpaA	117±33	68±19	141±27	150±19

Thylakoid membranes were isolated from cells grown in LL and the transferred to HL for 24 h. The activities of PSI and PSII were evaluated by measuring oxygen evolution resulting from electron transport from DCIP/ascorbic acid via PS I to MV and from H_2_O via PSII to phenyl-*p*-benzoquinone, respectively. Thylakoid membranes were added to the reaction mixture to the final concentration of 15 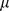
g/ml of chlorophyll. Data were generated by averaging three independent experiments on different days.

DCPIP- 2,6-Dichlorophenolindophenol; MV- Methyl viologen.

### RpaA Inactivation Reduces Monomeric PS I to Total PS II Ratio as Evaluated by Sucrose Gradient Ultracentrifugation, 77 K Fluorescence Emission Spectroscopy and Pigment Quantification

**Figure 6:**
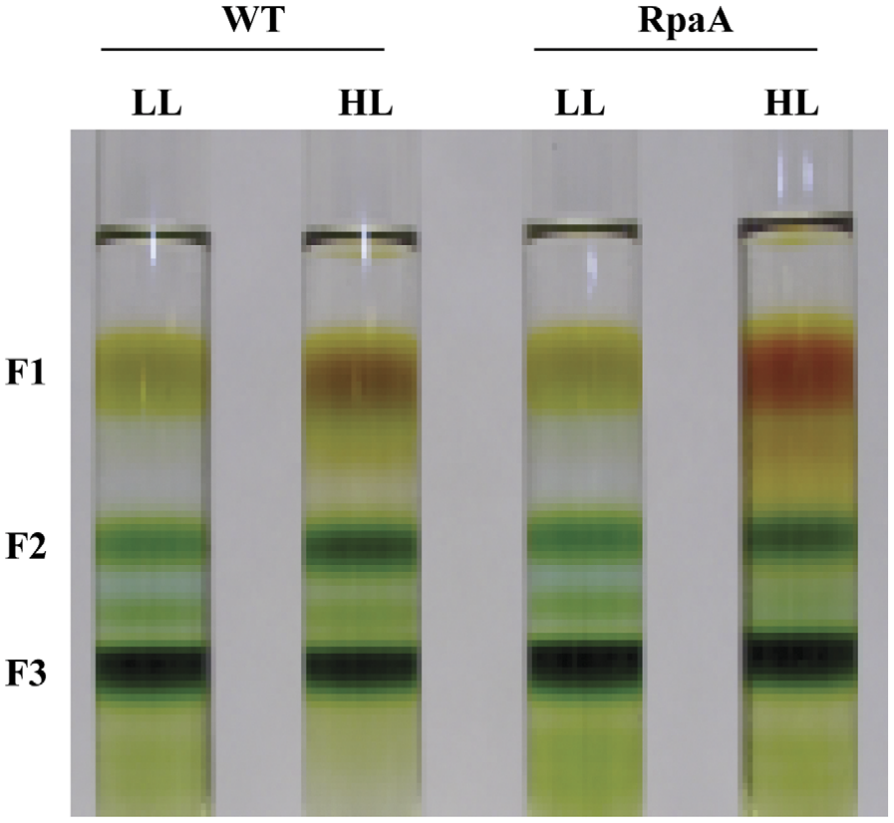
Sucrose gradient fractions of the thylakoid protein complexes from the WT and the RpaA inactivation mutant. Thylakoid membranes were isolated from the WT and the RpaA inactivation mutant grown in LL and HL (treated for 24 h). Thylakoid Protein complexes were separated by step sucrose-gradient ultracentrifugation.

Significant reduction in chlorophyll accumulation in the RpaA mutant instigated investigation of photosynthetic pigment complexes. Thylakoid membranes were prepared from both the WT and the mutant cells grown under normal growth light conditions, as well as in HL. The membranes isolated were solubilized to release photosynthetic pigment complexes, which were then separated by a 10%–40% step sucrose gradient ultracentrifugation (Fig. 6). Fraction 1 (F1) mainly constitutes free carotenoids and trace amounts of chlorophyll while the fraction 2 (F2) is a mixture of PS II and monomeric PS I. The fraction 3 (F3) is the PS I trimer. The F2 and F3 were collected and total chlorophyll per band of sucrose density gradient was measured (Table 4). F2 and F3 were further investigated by 77 K fluorescence and western blots.

**Table 4:** Total chlorophyll per band of sucrose density gradient.

Strain	Total chlorophyll per band of sucrose density gradient (µg)			
	F3; LL	F3; HL	F2; LL	F2; HL
WT	152±15	130±13	31±4	51±6
RpaA	154±17	135±19	32±6	40±5

Thylakoid membranes from cells grown in LL and treated by HL for 24 h were isolated, solubilized and fractionated by sucrose density gradient. A total of 200 µg chlorophyll was loaded on sucrose density gradient. Total chlorophyll content of fraction 2 (F2) and fraction 3 (F3) was determined. Data were generated by averaging three independent experiments on different days.

**Figure 7:**
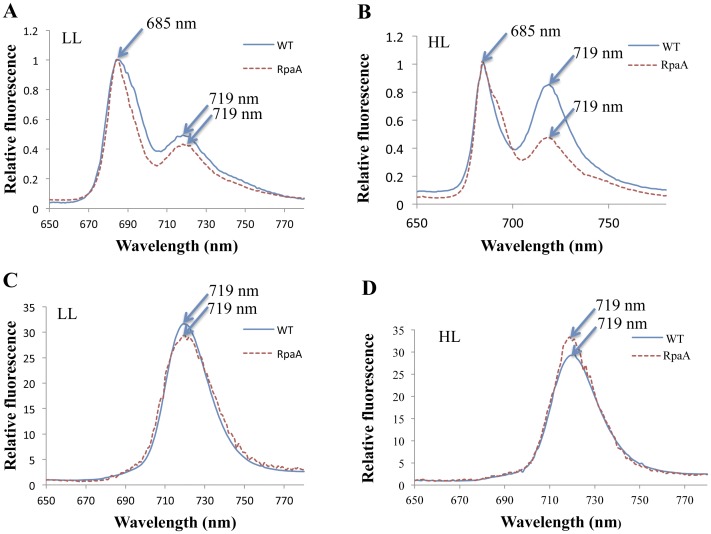
77 K fluorescence of sucrose gradient F2 and F3 of WT and the RpaA inactivation mutant. Thylakoid membranes from cells grown in LL and treated with HL for 24 h were isolated, solubilized and fractionated by sucrose gradient. The fluorescence emission spectra of the sucrose gradient fractions were recorded with an excitation wavelength of 430 nm at 77 K. All samples correspond to 15 µg of chlorophyll/ml. The spectra of F2 (PS II and monomeric PS I) were normalized at 685 nm; the spectra of F3 (PS I trimer) were normalized at 650 nm.

The pigment complexes (F2 and F3) separated by sucrose gradient ultracentrifugation were examined by 77 K fluorescence spectroscopy. The typical fluorescence emission spectra of F2 (the PS I monomer and PS II mixture) are shown in Figure 7A & B. The mutant exhibited a much lower *F_720_* and a higher *F_695_* fluorescence peak when normalized at *F_685_* under HL conditions. This indicates that the monomeric PS I to PS II ratio is lower in the mutant than in WT. The 77 K fluorescence of F3 (PS I trimers) (Fig. 7D) exhibited a slightly higher *F_720_* peak in the mutant than in WT on per chlorophyll or protein basis under HL condition, but the difference is slight. Therefore, inactivation of the RpaA does not affect the fluorescence yield of the PS I trimers.

### Western Blot Analysis Reveals that RpaA Inactivation Reduces Monomeric PS I and D1 Accumulation under HL Conditions

**Figure 8:**
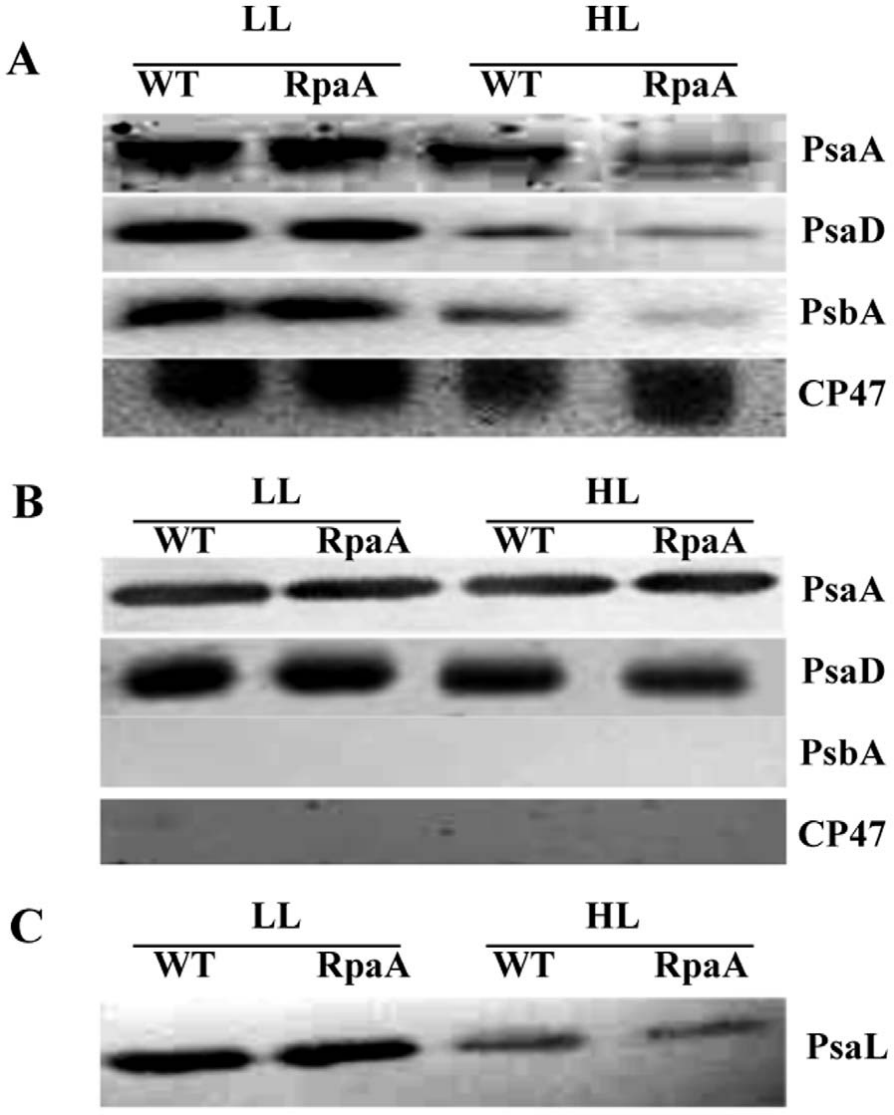
Immunoblot analysis of sucrose-gradient fraction 2 and 3 of WT and the RpaA inactivation mutant. **A**. F2, which contains PS II and monomeric PS I complexes, was collected and separated by a 12%–20% SDS-PAGE with 6 M urea. PsaA, PsaD and D1 were identified using corresponding polyclonal antibodies. **B**, The PsaA, PsaD, PsbA and CP47protein of the F3 (containing PS I trimers) was analyzed by Western blot using anti-PsaA, PsaD, PsbA and CP47 antibodies. **C**, The PsaL protein in thylakoid membranes was estimated using anti PsaL antibody.

We determined the content of PsaA (PS I-protein), PsbA or D1, and CP47 (subunits of PS II) in the photosynthetic pigment complexes separated by sucrose gradient to evaluate the potential regulatory role of RpaA on the photosynthetic protein complexes. 20 µg of total protein from F2 (monomeric PS I and PS II) were separated on 12% SDS-PAGE with 6 M urea, blotted onto nitrocellulose membranes, and probed with specific antibodies. The results are shown in Figure 8. There is dramatic reduction in the PsaA and PsaD contents of F2 in the mutant when compared to WT (Fig. 8A). The F3 contains exclusively PS I trimers free of PS II proteins in all of the samples (Fig. 8B), and all F3 samples contain similar amounts of PsaA or PsaD on either per chlorophyll or per protein basis. This suggests that inactivation of RpaA does not affect the characteristics of the PS I trimers. However, there appears to be a slightly higher concentration of the PS I trimers per volume in the mutant samples treated with HL (data not shown). This is consistent with the pigment data in Table 4. Therefore, the reduction of the monomeric PS I, as indicated by the significant reduction of PsaA and PsaD in F2, is likely the main reason for the reduction in total chlorophyll fluorescence from PS I in thylakoids or whole cells at 77 K.

Because PsaL is required for formation of the PS I trimers, we examined the PsaL content in the mutant and WT cells by western blots. As compared to LL grown cells (Fig. 8C), both the mutant and WT contain much less PsaL when cells were grown in HL. The PsaL content of the mutant is similar to that of WT in LL, and it is somewhat lower in the mutant than in WT under HL conditions. Therefore, the reduction of monomeric PS I in the mutant is not caused by an increase in PsaL.

We also observed a significant reduction in the D1 protein in the F2 of the mutant, but the CP47 level remained almost the same in the samples treated with HL for 24 hours (Fig. 8A). Because D1 is sensitive to oxidative stress, we estimated lipid peroxidation, a commonly accepted indicator of oxidative stress, in cells grown in LL or treated with HL for 24 or 48 hours by measuring the content of MDA. The results are shown in Table 5. The MDA levels are found to be similar for both WT and RpaA deletion strain in both LL and HL. No significant difference observed in MDA level in mutant than WT is suggestive of other damages, which might have outweighed lipid peroxidation and caused the striking phenotype in mutant. It also suggests that the RpaA inactivation affects accumulation of the D1 protein in HL, whose level is normally governed by the D1 turnover (degradation and *de novo* synthesis).

**Table 5:** Oxidative stress of WT and the RpaA mutant.

Strain	MDA (µM)		
	LL	24 Hrs	48 Hrs
WT	0.30±0.08	0.71±0.19	0.84±0.23
RpaA	0.32±0.12	0.78±0.24	0.88±0.27

Cells were grown in LL and HL for 48 h in BG-11 medium. Cells were lysed in the presence of 5 mM butylated hydroxytoluene to prevent sample oxidation during preparation. Concentrations of malondialdehyde (MDA) are presented in µM. The data were derived from three independent experiments.

## Discussion

Cyanobacteria respond to varying light conditions in a highly regulated manner, likely through highly sophisticated fabrics of their gene regulatory systems. Indeed, a relatively large number of regulatory genes are involved in acclimation and survival of *Synechocystis* sp. PCC 6803 [32]. It has been well documented that the transfer of cyanobacterial cells from low light (LL) to HL results in reduction of photosystem contents, particularly the PS I content [14]. As a result, the PS I to PS II ratio decrease when cells are transferred from LL to HL. The defect in the adjustment of PSI to PSII ratio could lead to the growth inhibition under HL [33]. The process by which the PS I to PS II ratio is regulated and orchestrated through the regulatory network is not well understood despite of active investigation in past years. The modulations in transcript abundance have been reported to be involved in the regulation of PS I content during acclimation to incident light [34]–[36]. *pmgA*, a novel gene, was identified and characterized as a specific regulator of the stoichiometry of photosystems in *Synechocystis* sp. PCC 6803 under HL conditions [13]. The *pmgA* mutant has defects in the transcriptional regulation of *psaAB* genes under HL conditions, and its inability to suppress PS I content under HL condition causes a higher electron transport rate which results in a higher photosynthesis rate. The regulatory gene *rpaA* has been shown to regulate the energy transfer from PBS to PS I [27] and serve as a circadian timing mediator [29]. In this work, we found that it regulates the accumulation of the monomeric PS I and the D1 protein under HL conditions.

The PS I complexes in cyanobacteria are organized in the thylakoid membrane preferentially as trimers [37], [38]. The amount of PS I trimers reduces in HL [10], [17]. However, the monomeric PS I to PS II ratio was observed to be higher in the *hli* deletion mutant and the *vipp1* deletion mutant [10], [16]. The decrease in PS I to PS II ratio may be attributed to a larger reduction in PS I than PS II. Since it has been observed that 90% of the chlorophyll is associated with PS I [39], chlorophyll plays an important role in modulating the PS I content under HL conditions in *Synechocystis* PCC 6803 [17]. The overall reduction in chlorophyll content per cell is generally interpreted as an indication of PS I content reduction. We observed ∼50% reduction in chlorophyll accumulation in the RpaA inactivation mutant as compared with WT when the cells were exposed to HL for 72 hours. This suggests that the RpaA inactivation caused a reduction in PS I. This is consistent with the 77 K fluorescence emission data. The spectra from the whole cell clearly showed reduced fluorescence from PS I in the mutant as compared to WT.

The PS I trimers and monomers constitute the major PS I pool in the thylakoid membranes. We analyzed these two types of PS I complexes (and PS II) by sucrose gradient ultracentrifugation. The 77 K fluorescence emission spectra from the PS I trimers (F3) remained almost the same as WT, suggesting that the RpaA inactivation does not affect the assembly or characteristics of the PS I trimers. In contrast, the fluorescence emission spectra of the F2, a mixture of PS I monomer and total PS II, revealed a striking difference between the mutant and WT: there is a significant reduction of the monomeric PS I to PS II ratio in the mutant as compared to WT (Fig. 7). Therefore, the reduced PS I to PS II ratio in the mutant is primarily caused by a reduction in the PS I monomer content. This is verified by western blot analysis. The mutant only retained ∼35% of PsaA and a significantly reduced amounts of PsaD in F2 as compared to WT under HL conditions, whereas the level of the PS I trimers in the mutant is comparable to that of WT as indicated by the amount of chlorophyll associated with PS I trimers (Table 4). Because the PsaL amount in the mutant is somewhat lower than that of WT in HL (Fig. 8C), it is clear that the reduction of the monomeric PS I in the RpaA inactivation mutant cannot be the result of an increase in PsaL, which might promote the assembly of PS I trimers, leading to a decrease in the amount of PS I monomers. Instead, we favor the interpretation that RpaA regulates the accumulation of monomeric PS I. So far, it is unknown how accumulation of PS I monomers and trimers is regulated. This is the first report that inactivation of a stress response regulator has specifically reduced monomeric photosystem I. It suggests that PS I monomers and PS I trimers can be regulated independently in response to regulatory clues or stimuli.

Another striking finding made in this work is that the D1 level in the mutant is reduced to 10% of WT level on an equal protein basis following HL exposure for 24 hours (Fig. 8A). However, neither the reduction in the total PS I to PS II ratio nor the reduction in the monomeric PS I to PS II ratio is as pronounced as the reduction in the D1 content; this suggests that the reduction of D1 seen in the mutant is not accompanied by the co-reduction of other PS II components. Indeed, no reduction of CP47 was observed in the RpaA inactivation mutant (Fig. 8A). Therefore, RpaA specifically regulates the accumulation or turnover of the D1 protein (but not other PS II proteins). It is well-established that PS II is the primary target of photoinhibition. In particular, the D1 protein undergoes rapid degradation following photoinhibition which is followed by *de novo* synthesis of D1 polypeptide and the incorporation of a new D1 copy into the PS II complex. Therefore, the striking reduction of D1 in the mutant following a 24-h HL exposure could be due to a reduced synthesis of D1 or an enhanced degradation due to oxidative stress. We favor the former possibility since there is no significant difference in the degrees of lipid peroxidation, a widely used indicator of oxidative stress, between WT and the RpaA inactivation mutant that were treated by HL for 24 or 48 hours (Table 5), despite the mutant exhibited growth defect after longer exposure to HL (Fig. 2D).

It is interesting to find that RpaA, a regulator of the circadian clock, also controls aspects of high light responses. RpaA is under the control of DspA, which has been implicated as a global regulator that controls high light and other stress responses [18], [30]. Like eukaryotic cells, cyanobacterial cells possess an endogenous “biological clock” that regulates biochemical or physiological processes in a roughly 24-hour cycle. In *Synechococcus elongatus* PCC 7942, almost all gene promoters are under the control of the circadian clock [29]. Circadian clocks seem to be more than patterns of gene expressions, but cell-signaling networks, which respond to other signal clues (e.g., environmental stresses), as well [40], [41]. The interaction between HL stress and the clock-signaling networks would allow the cells to adjust their circadian clock or gene expression patterns (transcriptionally or posttranscriptionally), permitting them to survive the stress.

Inactivation of RpaA impacted cell growth and fitness under HL conditions. The visible phenotype was readily observed when the mutant cells were grown on a solid BG11 plate (Fig. 2). The mutant cells retained less chlorophyll and appeared more orange in color. By contrast, there was no significant difference in the transcript levels of all of the genes that we examined: *psaA*, *pasB, psaL, psbA_2_*, *psbB, psbC, psbD,apcA, hliA*, *hliB*, *hliC*, *hliD* (Fig. 4).

Based on the data presented here, we propose that RpaA regulates the accumulation of the monomeric PS I and the D1 protein in HL (perhaps posttranscriptionally). This is the first report demonstrating that inactivation of a stress response regulator has specifically reduced the monomeric photosystem I. It suggests that PS I monomers and PS I trimers can be regulated independently in response to regulatory clues or stimuli. Such regulation by RpaA is critical for HL acclimation as the RpaA inactivation had a clear impact on the general growth and fitness of the cells under HL conditions.

## Materials and Methods

### Culture Growth Conditions

*Synechocystis* sp. PCC 6803 strains were grown at 30°C in BG-11 medium buffered with 5 mM *N*-tris(hydroxymethyl) methyl-2-aminoethanesulfonic acid (TES)-NaOH (pH 8.0). For photomixotrophic and photoheterotrophic growth, the medium was supplemented with 10 mM glucose. The culture was bubbled with CO_2_ under LL (50 µmol photon m^−2^ s^−1^) or HL (400 µmol photon m^−2^ s^−1^).

For growth on plates, 1.5% (w/v) Difco agar and 0.3% (w/v) sodium thiosulfate were added. The BG-11 media were supplemented with antibiotics appropriate for particular strains.

### DNA Manipulation and Mutant Construction

The *rpaA* gene was inactivated from WT *Synechocystis* sp. PCC 6803 by target mutagenesis and homoplasmic mutants were obtained. To inactivate the *rpaA* gene (*slr0115*), the 915-bp DNA fragment (corresponding to 100-bp upstream of the *slr0115* start codon to 87-bp downstream to stop codon) from *Synechocystis* sp. PCC 6803 genome was amplified. The primers used were 5′-GGAAACAGGGCGCTAATTTTCAG-3′ and 5′- TGGTCAAACTTAAATCAGTGA-CGG-3′. The DNA fragment covering the entire *slr0115* was cloned into the pGEMT vector. The *rpaA* gene was interrupted at *Sma*I site located 340-bp downstream of the coding sequence by inserting a 0.85-kb chloramphenicol resistance cartridge. The resulting plasmid, pGEMT-slr0115-cm^r^, was transformed into WT *Synechocystis* sp. PCC 6803. The transformants were selected by screening for resistance to 35 mg/L chloramphenicol in BG-11 medium. Transformants were restreaked onto a BG-11 plate containing chloramphenicol to get homozygous mutants. Segregation of inactivated *slr0115* was monitored by PCR, using genomic DNA obtained from the mutant.

### Oxygen Evolution

Cells were grown to mid-logarithmic phase and harvested by centrifugation at 5000×g for 5 min. The cells were then resuspended in fresh BG 11 medium to the concentration of 5 µg of chlorophyll/ml. Oxygen evolution was measured using a Clark-type electrode and illuminated with white light at 1000 µmol of photon m^−2^ s^−1^. Cultures were supplemented with 1 mM sodium bicarbonate for the determination of the whole chain photosynthetic electron transport activity.

Electron transport rates of PSI or PSII were estimated by measuring O_2_ consumption/evolution using a Clark-type electrode. The light intensity used was 500 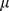
mol of photons m^−2^ s^−1^ white light. Thylakoid membranes were adjusted to a chlorophyll content of 15 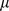
g/ml for all measurements. The PSI reaction mixture contains 40 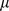
m MV, 5 mm NH_4_Cl, 2 mm ascorbic acid, 0.1 mm 2,6-dichlorophenolindophenol (DCPIP), 2 mm NaN_3_, 40 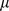
m 3-(3,4-dichlorophenyl)-1,1-dimethylurea (DCMU), 40 mm tricine (pH 7.5), and 100 mm sucrose. The reaction permits measurement of the electron transport rates from DCIP/ascorbic acid via PSI to MV; one oxygen molecule is consumed for each electron transport event. The PSII reaction mixture contains 5 mm NH_4_Cl, 4 mm K_3_FeCN_6_, 1 mm phenyl-*p*-benzoquinone, 40 mm tricine (pH 7.5), and 100 mm sucrose, which measures the electron transport rate from H_2_O via PSII to phenyl-*p*-benzoquinone; one oxygen molecule is produced for every four electrons transported. O_2_ evolution/consumption was followed for 3 min, and the rate was calculated accordingly.

### Lipid Peroxidation Assessment

Lipid peroxidation was assessed by measuring the amount of malondialdehyde (MDA) the decomposed products of polyunsaturated fatty acid peroxides. MDA were quantified using the BIOXYTECH® LPO-586™ kit (OXIS International Inc.) according to the manufacturer’s instructions. This assay is based on the reaction of a chromogenic reagent N-methyl-2-phenylindole with MDA at 45°C to yield a stable chromophore with maximal absorbance at 586 nm.

### Genomic DNA Preparation for PCR Analysis

A loopful of *Synechocystis* sp. PCC 6803 cells was suspended in 200 µl of Tris-EDTA buffer (pH 8.0) and transferred to a microcentrifuge tube with 200 µl of glass beads (0.1-mm diameter; Sigma). The cells were broken in a MiniBead Beater (Biospec Products) by two cycles of agitation at the low speed setting for 30 s. Cells were cooled on ice for 2 minutes prior to and after bead beating. Lysates were extracted with phenol-chloroform, and the DNA was precipitated, washed, and dried according to standard procedure [42]. The dry DNA pellet was dissolved in 150 µl milliQ water.

### Thylakoid Membrane Preparation and Fractionation of Membrane Protein Complexes

Thylakoid membranes were prepared as described [43] with some modifications. Briefly, cell pellets derived from cells grown to mid-logarithmic phase were resuspended in ice-cold thylakoid buffer (50 mM MOPS, pH 7.0, 0.4 mM Sucrose, 10 mM NaCl, 1 mM freshly made phenylmethylsulfonyl fluoride (PMSF). An equal volume of glass beads prewetted by thylakoid buffer was added to the cell suspension, and the cells were broken in a bead beater with an ice-jacketed sample chamber by six breakage cycles at full speed (30 s of bursts, followed by 5 min of chilling). The homogenate was centrifuged at 1,800×g for 10 min to remove unbroken cells, cellular debris, and glass beads. The membranes in supernatant were then pelleted by centrifugation at 50,000 g at 4°C for 60 min. After washing with 2 mM dodecyl maltoside to remove any remaining phycobilisomes, the membranes were washed twice and resuspended in thylakoid buffer to a chlorophyll *a* concentration of 1 mg/mL. The chlorophyll *a* concentration was estimated from the dimethylformamide extract by the formula developed by [44]:
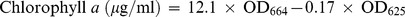



To fractionate membrane protein complexes, 150 µl of 10% dodecyl maltoside were added to the thylakoid membrane to achieve a detergent to chlorophyll ratio of 15 to 1. The thylakoid membrane was solubilized at 4°C for 30 min and was loaded onto 10% to 40% (w/w) step sucrose gradient and centrifuged at 160,000 g for 16 h at 4°C. Pigmented fractions were collected and stored at −80°C until use.

### Western Blot

SDS-PAGE was performed as described by [45]. Approximately 20 µg of membrane proteins were resolved by SDS-PAGE in a 12% polyacrylamide with 6 M urea. Polypeptides were electrophoretically transferred onto nitrocellulose membrane, and immunodetection of polypeptides was performed using commercial antibodies (Agrisera) except the anti PsaL antibodies (a kind gift from Dr Wu Xu from University of Louisana at Lafayette).

### Pigment Analysis

For qualitative analysis, 20–50 ml *Synechocystis* cells (OD_730_∼0.4–0.7) were harvested, and pigments were extracted from cell pellets by three successive extractions with methanol containing 0.1% (v/v) ammonium hydroxide. For quantitative determination of the pigment content of the cells, pigments were extracted in dimethylformamide (DMF). After centrifugation in a microcentrifuge at 4°C for 10 min, the supernatant was taken to quantify the pigment content photometrically. The chlorophyll concentration was calculated by the formula developed by [44] as described above.

### RT-PCR

Relative transcript levels of various genes were evaluated by the RT-PCR. Total RNA was isolated from 100 ml *Synechocystis* sp. PCC 6803 cultures as previously described [46]. Total RNA was treated with RNase free-DNase (Qiagen) to remove any trace of genomic DNA. Reverse transcription reaction was carried out using Superscript III enzyme (Invitrogen) using random primer. The resultant cDNA were amplified by PCR using specific primers for different genes. The PCR product was analyzed by 0.8% agarose gel electrophoresis.

### Chlorophyll Fluorescence Analysis and 77 K Fluorescence Spectroscopy

Chlorophyll fluorescence measurements were performed on a dual modulation kinetic fluorometer (dual PAM-100 system, Walz) as previously described [47]. Fluorescence emission spectra were recorded at 77 K using Fluromax-4 spectrofluorometer (Horiba Jobin YVON) at excitation wavelength of 430 nm and excitation and emission bandwidths of 5 and 1.5 nm, respectively, as previously described [10]. The chlorophyll concentration was adjusted to 15 µg/ml.
